# Variants in *ELL2* influencing immunoglobulin levels associate with multiple myeloma

**DOI:** 10.1038/ncomms8213

**Published:** 2015-05-26

**Authors:** Bhairavi Swaminathan, Guðmar Thorleifsson, Magnus Jöud, Mina Ali, Ellinor Johnsson, Ram Ajore, Patrick Sulem, Britt-Marie Halvarsson, Guðmundur Eyjolfsson, Vilhelmina Haraldsdottir, Christina Hultman, Erik Ingelsson, Sigurður Y. Kristinsson, Anna K. Kähler, Stig Lenhoff, Gisli Masson, Ulf-Henrik Mellqvist, Robert Månsson, Sven Nelander, Isleifur Olafsson, Olof Sigurðardottir, Hlif Steingrimsdóttir, Annette Vangsted, Ulla Vogel, Anders Waage, Hareth Nahi, Daniel F. Gudbjartsson, Thorunn Rafnar, Ingemar Turesson, Urban Gullberg, Kári Stefánsson, Markus Hansson, Unnur Thorsteinsdóttir, Björn Nilsson

**Affiliations:** 1 Hematology and Transfusion Medicine, Department of Laboratory Medicine, Lund University, BMC B13, SE-221 84 Lund, Sweden; 2 deCODE genetics, Sturlugata 8, IS-101 Reykjavik, Iceland; 3 Clinical Immunology and Transfusion Medicine, Laboratory Medicine, Office of Medical Services, Akutgatan 8, SE-221 85 Lund, Sweden; 4 The Laboratory in Mjodd, IS-109 Reykjavik, Iceland; 5 Department of Hematology, Landspitali, The National University Hospital of Iceland, IS-101 Reykjavik, Iceland; 6 Department of Medical Epidemiology and Biostatistics, Karolinska Institutet, SE-171 77 Stockholm, Sweden; 7 Department of Medical Sciences, Molecular Epidemiology and Science for Life Laboratory, Uppsala University, SE-751 85 Uppsala, Sweden; 8 Faculty of Medicine, University of Iceland, IS-101 Reykjavik, Iceland; 9 Hematology Clinic, Skåne University Hospital, SE-221 85 Lund, Sweden; 10 Section of Hematology, Sahlgrenska University Hospital, SE-413 45 Gothenburg, Sweden; 11 Center for Hematology and Regenerative Medicine, Karolinska Institutet, SE-171 77 Stockholm, Sweden; 12 Department of Immunology, Pathology and Genetics, Uppsala University, Rudbeck Laboratory, SE-751 05 Uppsala, Sweden; 13 Department of Clinical Biochemistry, Landspitali, The National University Hospital of Iceland, IS-101 Reykjavik, Iceland; 14 Department of Clinical Biochemistry, Akureyri Hospital, IS-600 Akureyri, Iceland; 15 Department of Haematology, University Hospital of Copenhagen at Rigshospitalet, Blegdamsvej 9, DK-2100 Copenhagen, Denmark; 16 National Research Centre for the Working Environment, Lersø Parkallé 105, DK-2100 Copenhagen, Denmark; 17 Department of Cancer Research and Molecular Medicine, Norwegian University of Science and Technology, Box 8905, N-7491 Trondheim, Norway; 18 Broad Institute, 7 Cambridge Center, Cambridge, Massachusetts 02142, USA

## Abstract

Multiple myeloma (MM) is characterized by an uninhibited, clonal growth of plasma cells. While first-degree relatives of patients with MM show an increased risk of MM, the genetic basis of inherited MM susceptibility is incompletely understood. Here we report a genome-wide association study in the Nordic region identifying a novel MM risk locus at *ELL2* (rs56219066T; odds ratio (OR)=1.25; *P*=9.6 × 10^−10^). This gene encodes a stoichiometrically limiting component of the super-elongation complex that drives secretory-specific immunoglobulin mRNA production and transcriptional regulation in plasma cells. We find that the MM risk allele harbours a Thr298Ala missense variant in an *ELL2* domain required for transcription elongation. Consistent with a hypomorphic effect, we find that the MM risk allele also associates with reduced levels of immunoglobulin A (IgA) and G (IgG) in healthy subjects (*P*=8.6 × 10^−9^ and *P*=6.4 × 10^−3^, respectively) and, potentially, with an increased risk of bacterial meningitis (OR=1.30; *P*=0.0024).

## Abstract (web summary)

Multiple myeloma is an incurable and fatal disease characterized by uninhibited growth of plasma cells in the bone marrow. Here, Swaminathan *et al.* conduct a genome-wide association study and identify a novel risk locus at *ELL2*, which encodes a key component of the super-elongation complex.

## Graphical abstract

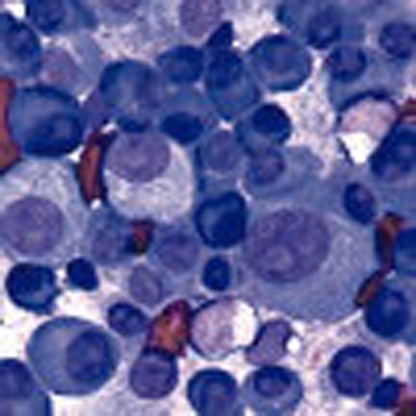

Multiple myeloma (MM) is characterized by an uninhibited, clonal growth of plasma cells in the bone marrow, producing a monoclonal immunoglobulin (‘M protein') that can be detected in peripheral blood^1^. According to the International Myeloma Working Group criteria, MM is defined by >10% monoclonal plasma cells in the bone marrow or >3 g M protein per 100 ml plasma. Characteristic symptoms include calcium elevation, renal insufficiency, anaemia and lytic bone lesions or osteoporosis. While survival can be extended, MM remains an incurable and fatal disease^2^. It is preceded by monoclonal gammopathy of unknown significance (MGUS)^3^,^4^, a common condition (3% of ≥50 year olds)^5^ defined as a clonal growth of plasma cells that does not yet satisfy the criteria for MM, but progresses to MM at a rate of ∼1% per year.

Since the 1970s, several authors have reported families with multiple cases of MM, including pedigrees suggesting Mendelian inheritance^6^,^7^. This century, systematic family-based studies, including in population-based registries, confirmed that first-degree relatives of patients with MM and MGUS have 2–4 times higher risk for MM, and a higher risk of certain other malignancies^8^,^9^,^10^,^11^,^12^. These data support the existence of MM risk alleles. Recent genome-wide association studies have identified eight common sequence variants that associate with MM, and account for an estimated 13% of the familial risk^13^,^14^,^15^. The molecular basis of inherited MM susceptibility is thus incompletely understood.

Here we report a genome-wide association study aimed at identifying DNA sequence variants that predispose for MM in Nordic populations. We identify a novel risk locus at the *ELL2* gene at 5q31 that encodes a key component of the super-elongation complex (SEC) that drives secretory-specific Ig mRNA production and transcriptional regulation in plasma cells. We also identify a promising association with the *TOM1-HMGXB4* locus at 22q13. We find that the *ELL2* MM risk allele harbours a Thr298Ala missense variant in an *ELL2* domain required for transcription elongation. Consistent with a hypomorphic effect, we find that the MM risk allele also associates with reduced levels of IgA and IgG in healthy subjects and, potentially, with an increased risk of bacterial meningitis.

## Results

### Genome-wide association study

To identify MM risk loci, we carried out a genome-wide association study based on one case–control data set from Sweden and Norway, and one from Iceland (Table 1). For the Swedish–Norwegian data set, variants identified by the 1,000 Genomes consortium were imputed into genotype data generated on Illumina single-nucleotide polymorphism (SNP) microarrays. For the Icelandic data set, variants were identified by whole-genome sequencing of 2,636 Icelanders^16^, and imputed into 104,220 Icelanders genotyped with Illumina SNP chips^17^,^18^. Using the Icelandic genealogy, we additionally calculated genotype probabilities for 294,212 relatives of the chip-typed individuals^16^.

We performed association testing in the Swedish–Norwegian and Icelandic data sets, and combined the results for 12.1 million variants that passed quality filtering. Two versions of the Icelandic case–control data were used for meta-analysis: one with MM patients only, and one that was expanded with non-IgM MGUS patients to increase power (Table 1). The latter is motivated because MM evolves from MGUS^3^,^19^, relatives of MGUS patients have increased MM risk^9^,^12^ and known MM risk alleles tend to associate with MGUS^11^. We replicated all known MM risk loci in both meta-analyses (Supplementary Fig. 1 and Supplementary Table 1; refs 13, 14, 15). Quantile–quantile analysis showed minimal *P* value inflation (genomic inflation factor *λ*=1.005–1.020; Supplementary Fig. 2).

**Figure 1:**
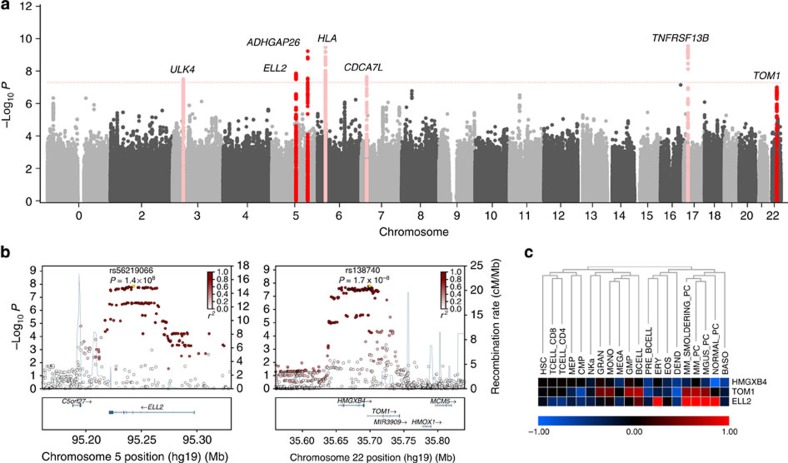
Identification of *ELL2* at 5q15 as a novel MM risk locus and *HMGXB4-TOM1* at 22q13 as a candidate MM risk locus. (**a**) Manhattan plot for the meta-analysis of the Swedish-Norwegian and Icelandic MM data sets for 12.1 million SNPs that passed quality filtering. Seven loci showed association with MM or MM+MGUS at meta-analysis *P*<5 × 10^−8^, including four known MM risk loci (pink) and three previously unknown loci at 5q15 (*ELL2*), 5q35 (*ARHGAP26*) and 22q13 (*HMGXB4* and *TOM1*) (red). The *x* axis indicates genomic position of the SNPs. The *y* axis indicates the –log_10_ of the combined *P* values. The dotted line indicates the threshold for genome-wide significance of meta-analysis *P*<5 × 10^−8^. The results shown were obtained with the MM+MGUS version of the Icelandic data. Similar results were obtained with the MM version (not shown). (**b**) Regional association plots of the novel risk locus at *ELL2* and the tentative risk locus at *HMGXB4*-*TOM1*. Positions and *P* values of SNPs indicated on the *x* and *y* axes, respectively. Degree of linkage disequilibrium with sentinel SNPs indicated in shades of red. Blue background curves indicate meiotic recombination rates. The signal at *ARHGAP26* was represented by an imputed rare variant that lost significance when genotyped directly, and was not investigated further. (**c**) Expression of *ELL2*, *TOM1* and *HMGXB4* in 20 different types of blood cells (Affymetrix microarrays). *ELL2* and *TOM1* are preferentially expressed in plasma cells. BASO, basophils; BCELL, B cells; CMP, common myeloid progenitors; EOS, eosinophils; ERY, erythroid progenitors; GMP, granulocyte–monocyte progenitors; HSCs, haematopoietic stem cells; MEGA, megakaryocytes; MEP, megakaryocyte–erythrocyte progenitors; MONO, monocytes; NEU, neutrophils; NK, natural killer cells; PC, CD138^+^ plasma cells; PRE-B, pre-B cells; TCELL, T cells.

Seven loci associated with MM or MM+MGUS at *P*<5 × 10^−8^ (calculated using logistic regression as described in Methods section). These included four known MM risk loci (Fig. 1a and Supplementary Table 1; refs 14, 15) and three previously unknown loci at 5q15 (*ELL2*), 5q31 (*ARHGAP26*) and 22q13 (*HMGXB4*-*TOM1*; Supplementary Table 2). Inclusion of the Icelandic MGUS cases strengthened the associations with 5q15 and 5q31. The signals at 5q15 (rs56219066; risk allele frequency (RAF) 71.1–73.2%) and 22q13 (rs138740; RAF 36.4–41.5%) were represented by common variants with moderate effects (odds ratio (OR)=1.20–1.39; Fig. 1a,b and Table 2), whereas the 5q31 signal (rs74735889; RAF ∼0.3%) was represented by an imputed rare variant that lost significance (OR=1.69; logistic regression *P*=0.014) when genotyped directly and was not investigated further. Examining the expression patterns of *ELL2*, *TOM1* and *HMGXB4* across different types of blood cells, we noted that *ELL2* and *TOM1* are preferentially expressed in normal and malignant plasma cells (Fig. 1c). Conditional analysis did not reveal any underlying independent association signals at the *ELL2* or *HMGXB4-TOM1* loci.

To validate the 5q15 and 22q13 loci, we genotyped an additional 586 MM cases and 2,111 controls from Sweden and Denmark for rs56219066 and rs138740 (Table 1). The rs56219066 SNP replicated in these samples (logistic regression *P*=0.0046) and reached genome-wide significance under Bonferroni correction when the discovery and replication sets were combined (meta-analysis *P*=9.6 × 10^−10^; Table 2). While rs138740 did not replicate, it remained borderline significant (meta-analysis *P*=5.7 × 10^−8^) when the discovery and replication sets were combined and we observed effects in the same direction as in the meta-analysis (OR=1.04–1.08; Table 2). Further validation in larger data sets is needed to confirm the 22q13 locus.

### ELL2 regulates RNA processing in plasma cells

The association with 5q15 was captured by numerous markers in strong linkage disequilibrium distributed across a ∼40-kb haplotype block in *ELL2* (elongation factor, RNA polymerase II, 2; previously eleven-nineteen lysine-rich leukaemia gene 2) (Fig. 1b). This gene encodes a stoichiometrically limiting component of the SEC^20^, which mediates rapid gene induction by suppressing transient pausing of RNA polymerase II activity along the DNA^21^. Strikingly, ELL2 and the SEC play an important role in the differentiation of mature B cells into plasma cells^22^,^23^. In mature and memory B cells, which express *ELL2* at a low level, *IGH-*mRNA is translated to membrane-bound Ig. In plasma cells, *ELL2* is highly expressed and helps RNA polymerase II find a promoter-proximal weak poly(A)-site that is essentially hidden in B cells. This causes *IGH*-mRNA to be translated to secreted Ig at a high rate^24^,^25^. B cell-lineage *ELL2* conditional knockout mice exhibit curtailed humoral responses to immunization, reduced numbers of plasma cells in the spleen and fewer antibody-producing cells in the bone marrow. Plasma cells isolated from these mice show a paucity of secreted IgH and a distended endoplasmic reticulum^26^. Silencing of *ELL2* in mouse plasmacytoma cell lines using RNA interference decreases the ratio of secreted versus membrane-encoding Ighg2b transcripts^27^. RNA sequencing studies suggest that, in addition to the *IGH*-mRNA, *ELL2* influences the processing of ∼12% of transcripts expressed in plasma cells, including those of the plasma cell survival receptor *Tnfrsf17* (B-cell maturation antigen (BCMA))^22^,^26^ and the *MYC* oncogene^28^.

**Figure 2:**
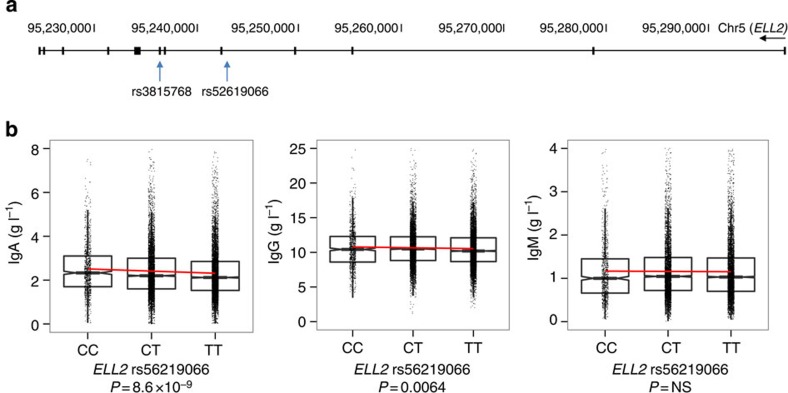
The *ELL2* MM risk allele harbours a Thr298Ala missense variant, and the sentinel SNP rs56219066 is associated with reduced Ig levels. (**a**) Schematic representation of the *ELL2* gene showing the location of the sentinel SNP rs56219066 in intron 4 and the correlated variant rs3815768 in exon 7, which causes a Thr298Ala substitution in an *ELL2* domain required for transcription elongation. (**b**) We analysed blood IgA, IgG and IgM levels from 24,279, 21,981 and 20,413 Icelandic individuals without MM or MGUS. We found a significant association between IgA and IgG levels and the *ELL2* risk allele (log-linear regression *P* values shown). Compared with rs56219066C homozygotes, rs56219066T heterozygotes and homozygotes show 5.2 and 10.1% lower IgA and 2.6 and 5.1% lower IgG, respectively. We observed similar effects for IgA and IgG in an independent set of 1,012 Swedish blood donors (Supplementary Fig. 3). Boxes indicate medians and the first and third quartiles. Whiskers indicate first and third quartiles 1.5 times the interquartile range or the minimum/maximum values. Notches indicate confidence intervals around the median. NS, not significant.

To characterize the *ELL2* risk allele, we analysed SNP and gene expression profiles from peripheral blood (eight data sets totalling 9,087 samples) and lymphoblastoid cell lines (two data sets totalling 1,188 samples). We did not detect any risk allele-associated effect on *ELL2* expression (not shown). Because we did not have access to expression data from plasma cells from genotyped individuals, we could not exclude a plasma cell-specific effect on *ELL2* expression. Among the associated variants, however, we identified a Thr298Ala missense variant in *ELL2* exon 7 (rs3815768) in tight linkage disequilibrium (*D′*/*r*^*2*^=1.00/0.957) with the sentinel SNP rs56219066 in intron 4 (Fig. 2a). The missense variant is located at the end of a ELL2 domain required for transcription elongation^29^.

### The *ELL2* risk allele associates with decreased IgA and IgG

**Figure 3:**
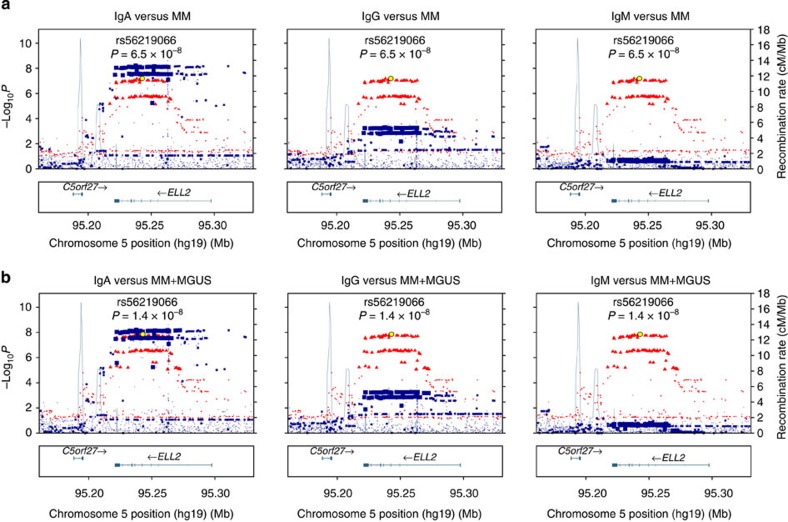
The *ELL2* haplotype that predisposes for MM is identical to the *ELL2* haplotype that influences IgA and IgG levels in healthy subjects. In addition to the sentinel SNP rs56219066, the association between 5q15 identified in the MM and MM+MGUS meta-analyses was captured by numerous markers in strong linkage disequilibrium located in a ∼40-kb haplotype block in *ELL2*. To verify that the *ELL2* haplotype associating with MM and MM+MGUS is identical to the *ELL2* haplotype associating with Ig levels, we tested for association between each available SNP in the *ELL2* region and IgA, IgG and IgM levels using the Icelandic Ig data set. We overlaid the log-linear regression *P* values for association with Ig levels with the meta-analysis *P* values obtained for the same SNPs for association with MM and MM+MGUS: (**a**) log-linear regression *P* values for association with IgA (red; left), IgG (red; middle) and IgM (red; right) overlaid on logistic regression *P* values for association with MM (blue); (**b**) corresponding results for MM+MGUS. The *x* axes indicate chromosomal positions. The *y* axes indicate −log_10_ *P* values. Sizes of markers reflect degree of association with MM or MM+MGUS. As shown, all SNPs in the ∼40-kb haplotype block associating with MM or MM+MGUS associate with IgA and, to a lesser extent, with IgG. We did not observe any association with IgM. Taken together, *ELL2* SNPs associating with MM and MM+MGUS associate with IgA and IgG and vice versa, further supporting that the *ELL2* haplotype that predisposes for MM also influences Ig levels.

Because of the recent mouse studies implicating *ELL2* in the production of secreted Igs, we tested for associations with blood Ig levels in 20,413–24,279 Icelanders without MM or MGUS. Risk allele carriers showed lower IgA (log-linear regression *P*=8.6 × 10^−9^) and IgG levels (log-linear regression *P*=6.4 × 10^−3^; Fig. 2b). The *ELL2* haplotype associating with IgA and IgG levels was identical to the haplotype associating with MM (Fig. 3). Compared with rs56219066C homozygotes, rs56219066T heterozygotes and homozygotes showed 5.2 and 10.1% lower IgA and 2.6 and 5.1% lower IgG, respectively (Fig. 2b). We observed similar effects in an independent set of 1,012 Swedish blood donors (Supplementary Fig. 3). The risk allele does not associate with IgM levels. These results, together with the reduced Ig levels in the *ELL2* conditional knockout mice^26^, suggest that the MM risk variant reduces, rather than enhances, the function of ELL2 in plasma cells.

### The *ELL2* risk allele associates with bacterial meningitis

Finally, to test whether the *ELL2* risk allele affects the risk of other diseases and traits, we screened deCODE's databases harbouring about 400 independent and uncorrelated diseases and quantitative traits. While we did not find any association with other B-lymphoid proliferative or malignant diseases apart from MGUS (OR=1.19; logistic regression *P*=0.0018), we observed associations between rs56219066T and lower total serum protein levels (*n*=20,100; log-linear regression *P*=0.0014; *β*= −0.035) as previously reported for rs3777200 in *ELL2* (*D*′/*r*^2^=1.00/0.96 with rs56219066; ref. 30), and an increased risk of bacterial meningitis (*n*=512; OR=1.30; logistic regression *P*=0.0024). The meningitis risk could be mediated through the reduced IgA and IgG levels.

## Discussion

We have identified a previously unknown MM risk locus at 5q15 (*ELL2*) and a promising MM risk locus at 22q13 (*HMGXB4*-*TOM1*). Neither of these loci has been previously associated with MM or other lymphoid malignancies. The identified risk variants are common and their estimated effect sizes are similar to those of previously identified MM risk variants^13^,^14^,^15^.

While the mechanisms that promote development of MM await further exploration, our findings indicate that the *ELL2* risk allele affects plasma cell function. The fact that *ELL2* regulates mRNA processing in plasma cells is compelling, as is the reduction of IgA and IgG levels associated with the risk allele. While the altered Ig levels as such are unlikely to be the MM-predisposing event (as other variants in the Icelandic data that alter Ig levels do not predispose for MM; not shown), these changes could reflect a hypomorphic effect on the SEC that affects mRNA processing broadly, which could predispose for malignant transformation.

Furthermore, the lower Ig levels could make *ELL2* risk allele carriers susceptible to infections. The potential association with meningitis is therefore intriguing. While these carriers are certainly not severely immunodeficient (because the allele is common), it is well known that various types of limited Ig deficiency (for example, IgG2 and IgG3 subclass deficiency) confer an increased incidence of infections, including with *Neisseria meningitidi*s and other meningitis pathogens. Future studies will uncover the role of *ELL2* in haematological malignancies and immune response.

## Methods

### Study populations

For the Swedish-Norwegian discovery sample set, we obtained 1,668 and 157 samples from the Swedish National Myeloma Biobank (Skåne University Hospital, Lund, Sweden) and the Norwegian Biobank for Myeloma (Trondheim, Norway), respectively. The samples were banked between 2003 and 2013. In addition, we obtained SNP microarray profiles of population-based controls from previous studies of twins (*n*=9835; TWINGENE, http://ki.se/sites/default/files/twingene_gwas_basic_info.pdf) and schizophrenia^31^ (*n*=3,754). From TWINGENE, we only used one individual from each pair of twins. After this filtering, a total of 10,704 controls were available for further analysis.

For the Icelandic discovery sample set, we identified from the nationwide Icelandic Cancer Registry all patients diagnosed with MM (ICD-10 code C90) in Iceland from 1955 to 2013 were identified and used in the association studies (*n*=480). To identify MGUS cases, information on the detection of an M protein on serum protein electrophoresis was gathered from 1955 to 2005 from Landspitali University Hospital and the Icelandic Medical Center Laboratory in Mjodd. A total of 251 cases of non-IgM MGUS were identified and used in the analysis.

For replication, we obtained 223 MM cases from the Swedish National Myeloma Biobank and 363 MM cases from the University Hospital of Copenhagen. As controls for the respective replication sets, we used 1,285 randomly ascertained Swedish blood donors and 826 randomly ascertained individuals from Denmark and Skåne county, Sweden (the southernmost part of Sweden next to Denmark). All samples were collected subject to ethical approval (Lund University Ethical Review Board, 2013/54; Icelandic Data Protection Authority, 2001010157; and National Bioethics Committee 01/015) and informed consent. No individuals were approached solely for the purpose of this study.

### Analysis of Swedish and Norwegian samples

Samples were genotyped on Illumina OmniExpress-Exome and OmniExpress microarrays. For analysis, we used the OmniExpress SNPs, which are recorded by both array types. We excluded SNPs showing >5% missing data, significant deviation from Hardy–Weinberg equilibrium (*P*<1 × 10^−1^ in controls; *P*<1 × 10^−1 × ^ in cases), or discrepancies in allele frequency between genotyping batches (*P*<5 × 10^−8^; *χ*^2^-test). We excluded samples showing >5% missing data or excess heterozygosity (>3 s.d.'s), and samples from closely related individuals (proportion identity-by-descent 
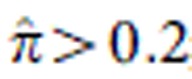
; calculated using SNPs with pairwise *r*^2^<0.2 using PLINK, after removing regions of high linkage disequilibrium^32^,^33^). After filtering, 542,599 SNPs, 1,714 cases and 10,391 controls remained. Unobserved genotypes were imputed using phased haplotypes from the Phase I (b37) release of the 1,000 Genomes Project^34^ ( http://www.1000genomes.org). Association testing was performed using logistic regression under an additive genetic model. To avoid artefacts of cryptic population stratification, we included five principal components of the identity-by-state matrix that were found to increase the genomic inflation factor *λ* (ref. 35) in the regression. The analyses were done with SHAPEIT2 (ref. 36; ( https://mathgen.stats.ox.ac.uk/genetics_software/shapeit/shapeit.html), IMPUTE2 (ref. 37; https://mathgen.stats.ox.ac.uk/impute/impute_v2.html) and SNPTEST^38^ ( https://mathgen.stats.ox.ac.uk/genetics_software/snptest/snptest.html).

### Analysis of Icelandic samples

Samples were genotyped using Illumina microarrays. The whole genomes of 2,636 Icelanders were sequenced using Illumina technology to a mean depth of at least 10 × (median 20 × ), including 909 to a mean depth of at least 30 × (ref. 39). A total of 35.5 million autosomal SNPs and indels were identified using the Genome Analysis Toolkit version 2.3.9 (ref. 40). We used information about haplotype sharing to improve variant genotyping, taking advantage of the fact that all sequenced individuals had also been chip-typed and long-range phased^17^. Variants were annotated using Ensembl release 72 and Variant Effect Predictor (VEP) version 2.8 (ref. 41). The 35.5 million sequence variants found and genotyped by whole-genome sequencing were then imputed into 104,220 Icelanders who had been genotyped using Illumina chips. In addition, using the Icelandic genealogy, we calculated genotype probabilities for 294,212 untyped individuals who are close relatives of the chip-typed individuals born after 1880 (Gudbjartsson *et al.* 39). Including this increases the power to detect associations with all diseases where ungenotyped cases are available. Logistic regression was used to test for association between SNPs and disease, treating disease status as the response and genotype counts as covariates. Other available individual characteristics that correlate with disease status were also included in the model as nuisance variables. These characteristics were as follows: sex, county of birth, current age or age at death (first and second order terms included), blood sample availability for the individual and an indicator function for the overlap of the lifetime of the individual with the time span of phenotype collection (described in detail below). The control set selected for each case group can thus be different after matching for the nuisance variables (Gudbjartsson *et al.* 39). Correction for familial relatedness was carried out using the method of genomic control by dividing the corresponding *χ*^2^-statistic by 1.04 and 1.02 for MM and MM+MGUS, respectively.

### Meta-analysis

We performed association testing in each discovery set separately and combined the results for 12.1 million variants that were shared by the Icelandic and 1,000 Genomes whole-genome sequencing data. These variants passed the quality thresholds applied: minor allele frequency >0.1%, imputation information value >0.8 and consistent frequency between the two sample sets. The meta-analysis was done using METAL^42^ ( http://www.sph.umich.edu/csg/abecasis/metal) with a fixed effect model. Conditional association analysis with respect to rs56219066 and rs138740 using SNPTEST^38^ did not reveal any other underlying, independent signals.

### Gene expression analysis in haematopoietic cell types

To characterize gene expression patterns of *ELL2*, *TOM1* and *HMGXB4*, we used microarray data from the Gene Expression Omnibus ( http://www.ncbi.nlm.nih.gov/geo). These data included gene expression profiles of different types of blood cells from normal haematopoiesis (*n*=211; accession no. GSE24759 (ref. 43)), plasma cells from patients with MM (*n*=1,285; accession nos. GSE15695 (ref. 44) GSE4581, GSE19784 (ref. 45) and GSE26760 (ref. 46)), and plasma cells from patients with MGUS, patients with smouldering MM and healthy bone marrow donors (*n*=78; accession no. GSE5900 (ref. 47)). All data were generated on Affymetrix U133A and Av2 microarrays and quantile-normalized to a log-normal distribution.

### Genotyping and association analysis of replication samples

The Swedish and Danish replication samples were genotyped by quantitative PCR for *ELL2* rs56219066 (Taqman custom assay AHCTDL6), *ELL2* rs3815768 (Taqman assay C_22272652_30) and *TOM1* rs138726 (*D*′/*r*^2^=1/0.997 with rs138740; Fluidigm SNP type assay GTA0072445). Association analysis for the replication sets was done using NEMO^48^ assuming a multiplicative risk model. Results for the discovery and replication cohorts were combined using a Mantel–Haenszel fixed effect model. Heterogeneity in the effect estimate was tested assuming that the estimated ORs for different groups followed a log-normal distribution using a likelihood ratio *χ*^2^-test with degrees of freedom equal to number of groups compared minus one.

### Association of the risk alleles with gene expression

To test for associations between identified risk variants and the expression of nearby genes, we analysed SNP and gene expression microarray data generated from peripheral blood samples (eight data sets totalling 973 individuals from the Icelandic population^49^ and 8,086 individuals of other European populations^50^) and lymphoblastoid cell lines (two data sets totalling 1,188 samples^51^,^52^). Gene expression in the Icelandic data set was quantified as the mean log_10_ expression ratio compared with pooled reference RNA samples, and regressed against the number of risk alleles carried, age, gender, relatedness and differential white blood cell counts.

### Association of *ELL2* allele with Ig levels

To screen for associations between the identified MM risk allele at *ELL2* and Ig levels, we used IgA, IgG and IgM measurements from 24,279, 21,981 and 20,413 individuals from the Icelandic population, respectively. Subjects diagnosed with MM or MGUS were not included in this data set. Ig levels adjusted for age, sex and site were tested for association with imputed genotypes using generalized log-linear regression^16^. Individuals diagnosed with MM or MGUS were excluded. In addition, we used IgA, IgG and IgM data from 1,012 Swedish blood donors (Clinical Immunology and Transfusion Medicine, Lund, Sweden) previously genotyped for *ELL2* rs17085249 (*D*′/*r*^2^=1/0.957 with rs56219066 and *D*′/*r*^2^=1/1 with rs3815768, Fluidigm SNP type assay GTA0072447). For the latter samples, we used Pearson correlation for association testing. All Ig measurements were done at certified clinical laboratories in Iceland and Sweden.

### Association of the *ELL2* risk allele with other traits

The deCODE Genetics phenotype database contains medical information on diseases and traits obtained through collaboration with specialists in each field. This includes information on cardiovascular diseases (myocardial infarction, coronary arterial disease, peripheral arterial disease, atrial fibrillation, sick sinus syndrome and stroke), metabolic disorders (obesity, diabetes and metabolic syndrome), psychiatric disorders (schizophrenia, bipolar disorder, anxiety and depression), addictions (nicotine and alcohol), inflammatory diseases (rheumatoid arthritis, lupus and asthma), musculoskeletal disorders (osteoarthritis, osteoporosis), eye diseases (glaucoma), kidney diseases (kidney stones and kidney failure) and 29 types of cancer. Anthropometric measures have also been collected through several of these projects. Routinely measured traits from patient workups (sodium, potassium, bicarbonate, calcium, phosphate, creatinine, blood cell counts, haemoglobin, haematocrit, Igs, iron, vitamins, lipids and more) were obtained from the Landspitali University Hospital, Reykjavik, and the Icelandic Medical Center Laboratory in Mjodd (Laeknasetrid), Reykjavik, in addition to more specific hormonal measures (adrenal, thyroid and sex hormones). The number of independent and uncorrelated secondary traits tested for association with rs56219066 amounts to 400.

## Additional information

**How to cite this article:** Swaminathan, B. *et al.* Variants in *ELL2* influencing immunoglobulin levels associate with multiple myeloma. *Nat. Commun.* 6:7213 doi: 10.1038/ncomms8213 (2015).

## Supplementary Material

Supplementary Information Supplementary Figures 1-3 and Supplementary Tables 1-2

## Figures and Tables

**Table 1:** Study populations.

	***N***	**Per cent male**	**Age at diagnosis (years±s.d.)**	**Genotyping method**
*Discovery samples*				
Sweden and Norway				
Cases	1,714	57.1%	67.5**±**11.4	Illumina OmniExpress-Exome^1^
Controls	10,391	51.5%	—	Illumina OmniExpress^1^
Iceland (MM)				
Cases	480	47.9%	71.2±10.0	Illumina microarrays (*n*=174); familially imputed (*n*=306)^2^
Controls	212,164	48.9%	—	Illumina microarrays (*n*=82,742); familially imputed (*n*=129,422)^2^
Iceland (MM+MGUS)				
Cases	731	49.5%	71.0**±**12.4	Illumina microarrays (*n*=332); familially imputed (*n*=399)^2^
Controls	283,999	48.8%	—	Illumina microarrays (*n*=90,568); familially imputed (*n*=193,431)^2^
				
*Replication samples*				
Sweden				
Cases	223	—	—	Selected SNPs
Controls	1,285	—	—	Selected SNPs
Denmark				
Cases	363	—	—	Selected SNPs
Controls	826	—	—	Selected SNPs

MGUS, monoclonal gammopathy of unknown significance; MM, multiple myeloma; SNP, single-nucleotide polymorphism.

^1^Imputed using whole-genome sequence data from 1,000 Genomes.

^2^Imputed using whole-genome sequence data from 2,636 Icelanders.

**Table 2:** Association of sequence variants in or near *ELL2* and *TOM1*.

**Populations**	**EAF**	**MM**		**MM+MGUS**	
		**OR (95% CI)**	***P*** **value**	**OR (95% CI)**	***P*** **value**
*ELL2*—rs56219066-T					
*Discovery*					
Sweden/Norway	0.732	1.20 (1.11–1.32)	3.8 × 10^−5^	1.20 (1.11–1.32)	3.8 × 10^−5^
Iceland	0.711	1.39 (1.17–1.64)	1.1 × 10^−4^	1.32 (1.15–1.51)	3.9 × 10^−5^
Combined		1.23 (1.14–1.33)	6.5 × 10^−8^	1.23 (1.15–1.33)	1.4 × 10^−8^
*Replication*					
Denmark (363/826)	0.732	1.28 (1.04–1.57)	0.017	1.28 (1.04–1.57)	0.017
Sweden (223/1285)	0.735	1.30 (1.03–1.64)	0.030	1.30 (1.03–1.64)	0.030
Combined		1.29 (1.08–1.54)	0.0046	1.29 (1.08–1.54)	0.0046
Combined discovery and replication		1.25 (1.16–1.34)	9.6 × 10^−10^	1.24 (1.16–1.33)	2.2 × 10^−10^
					
TOM1—rs138740-C					
*Discovery*					
Sweden/Norway	0.364	1.20 (1.11–1.30)	2.4 × 10^−6^	1.20 (1.11–1.30)	2.4 × 10^−6^
Iceland	0.415	1.26 (1.09–1.46)	0.0017	1.15 (1.03–1.30)	0.015
Combined		1.22 (1.13–1.30)	1.7 × 10^−8^	1.19 (1.11–1.27)	1.3 × 10^−7^
*Replication*					
Denmark (352/815)	0.360	1.08 (0.90–1.30)	0.39	1.08 (0.90–1.30)	0.39
Sweden (235/1285)	0.364	1.04 (0.85–1.26)	0.72	1.04 (0.85–1.26)	0.72
Combined		1.06 (0.93–1.21)	0.38	1.06 (0.93–1.21)	0.38
Combined discovery and replication		1.18 (1.11–1.25)	5.7 × 10^−8^	1.16 (1.10–1.23)	2.7 × 10^−7^

Abbreviations: CI, confidence interval; EAF, effect allele frequency; MGUS, monoclonal gammopathy of unknown significance; MM, multiple myeloma; OR, odds ratio.

Association results for *ELL2* rs56219066 and *TOM1* rs138740 in the discovery samples from Sweden, Iceland and Norway and in the replication samples from Sweden and Denmark. Logistic regression and meta-analysis *P* values indicated.
