# Supplementary material for: Variants in ELL2 influencing immunoglobulin levels associate with multiple myeloma
Source: Nat Commun. 2015 May 26;6:7213. doi: 10.1038/ncomms8213 (PMC4455110; doi:10.1038/ncomms8213)
Supplement: Supplementary Information — Supplementary Figures 1-3 and Supplementary Tables 1-2 [file ncomms8213-s1.pdf]

Supplementary Figure 1

a

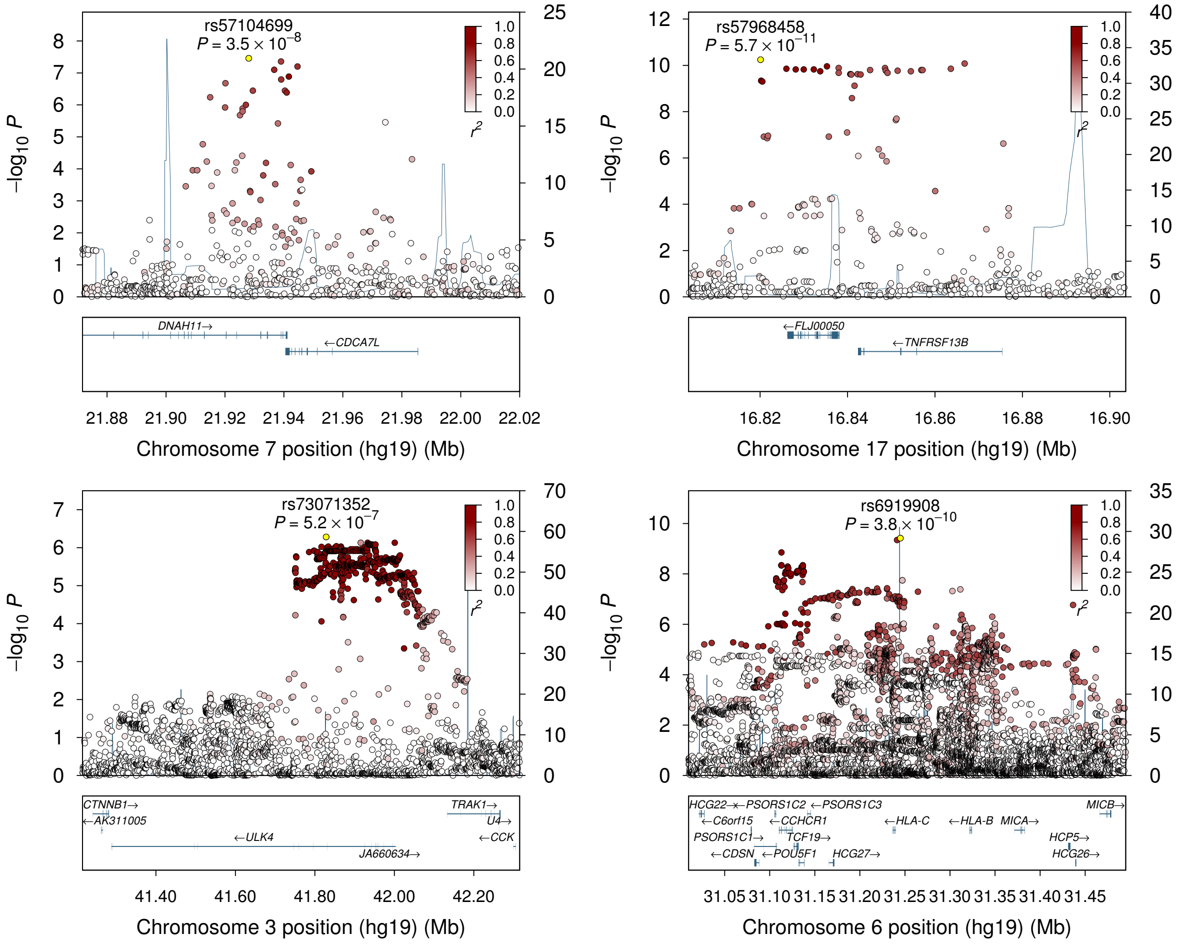

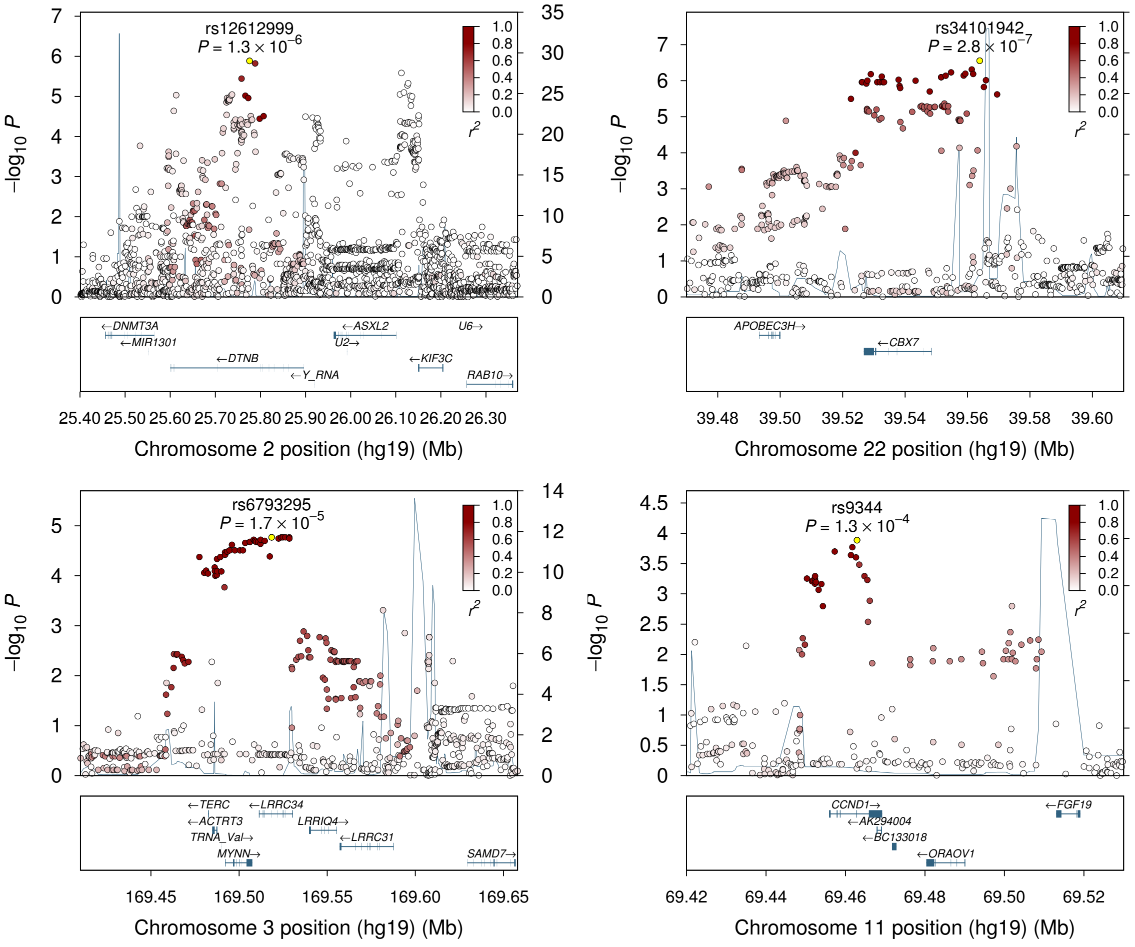
rs12612999
P = 1.3 × 10−6
−log10 P
r2
←DNMT3A
←MIR1301
←DTNB
←Y_RNA
←ASXL2
U2→
←KIF3C
U6→
RAB10→
Chromosome 2 position (hg19) (Mb)
rs34101942
P = 2.8 × 10−7
APOBEC3H→
←CBX7
Chromosome 22 position (hg19) (Mb)
rs6793295
P = 1.7 × 10−5
←TERC
←ACTRT3
TRNA_Val→
MYNN→
←LRRC34
LRRIQ4→
←LRRC31
SAMD7→
Chromosome 3 position (hg19) (Mb)
rs9344
P = 1.3 × 10−4
CCND1→
←AK294004
←BC133018
←ORAOV1
←FGF19
Chromosome 11 position (hg19) (Mb)

b

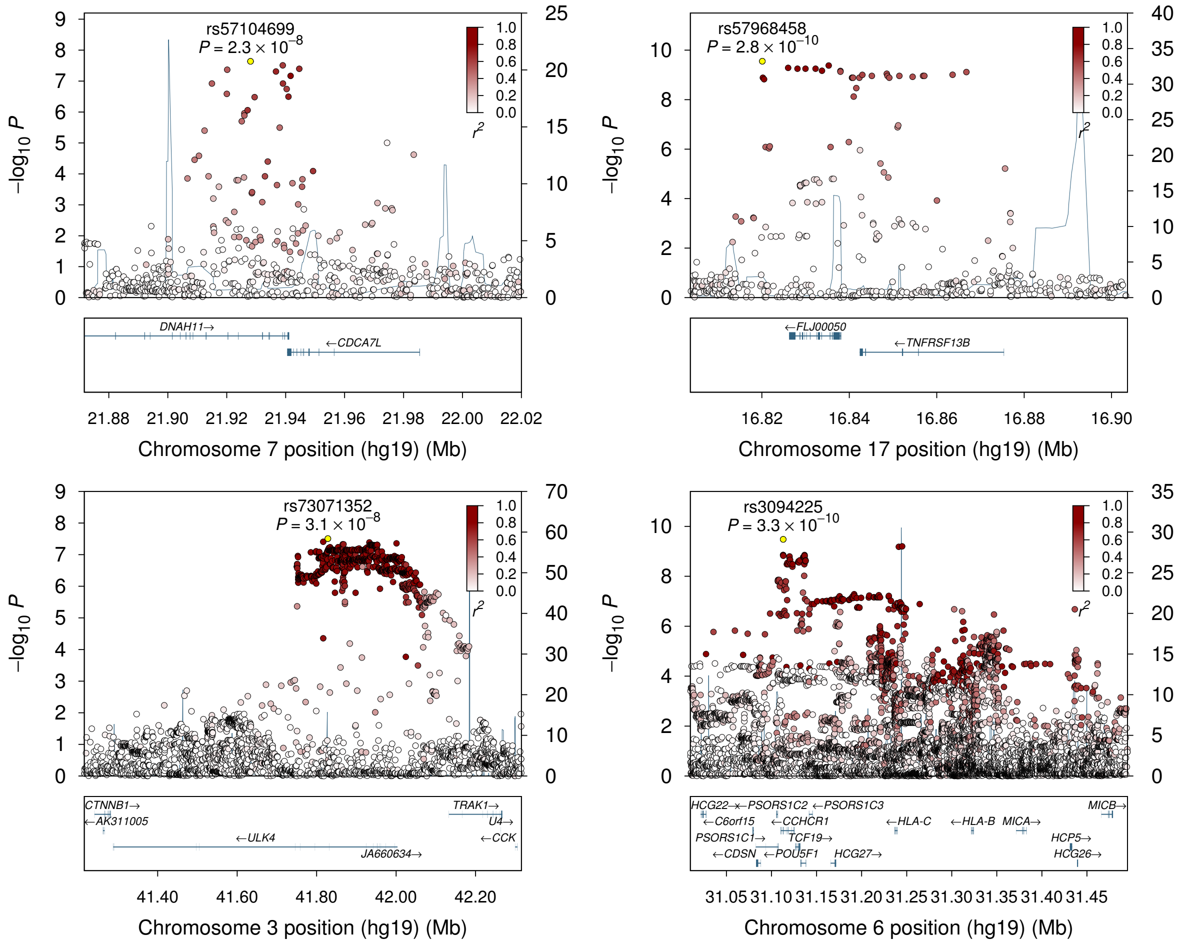
rs57104699
P = 2.3 × 10−8
DNAH11→
←CDCA7L
Chromosome 7 position (hg19) (Mb)
rs57968458
P = 2.8 × 10−10
←FLJ00050
←TNFRSF13B
Chromosome 17 position (hg19) (Mb)
rs73071352
P = 3.1 × 10−8
CTNNB1→
←AK311005
←ULK4
JA660634→
TRAK1→
U4→
←CCK
Chromosome 3 position (hg19) (Mb)
rs3094225
P = 3.3 × 10−10
HCG22→
←PSORS1C2
←PSORS1C3
←C6orf15
←CCHCR1
PSORS1C1→
TCF19→
←CDSN
←POU5F1
HCG27→
←HLA-C
←HLA-B
MICA→
MICB→
HCP5→
HCG26→
Chromosome 6 position (hg19) (Mb)
−log10 P
r2

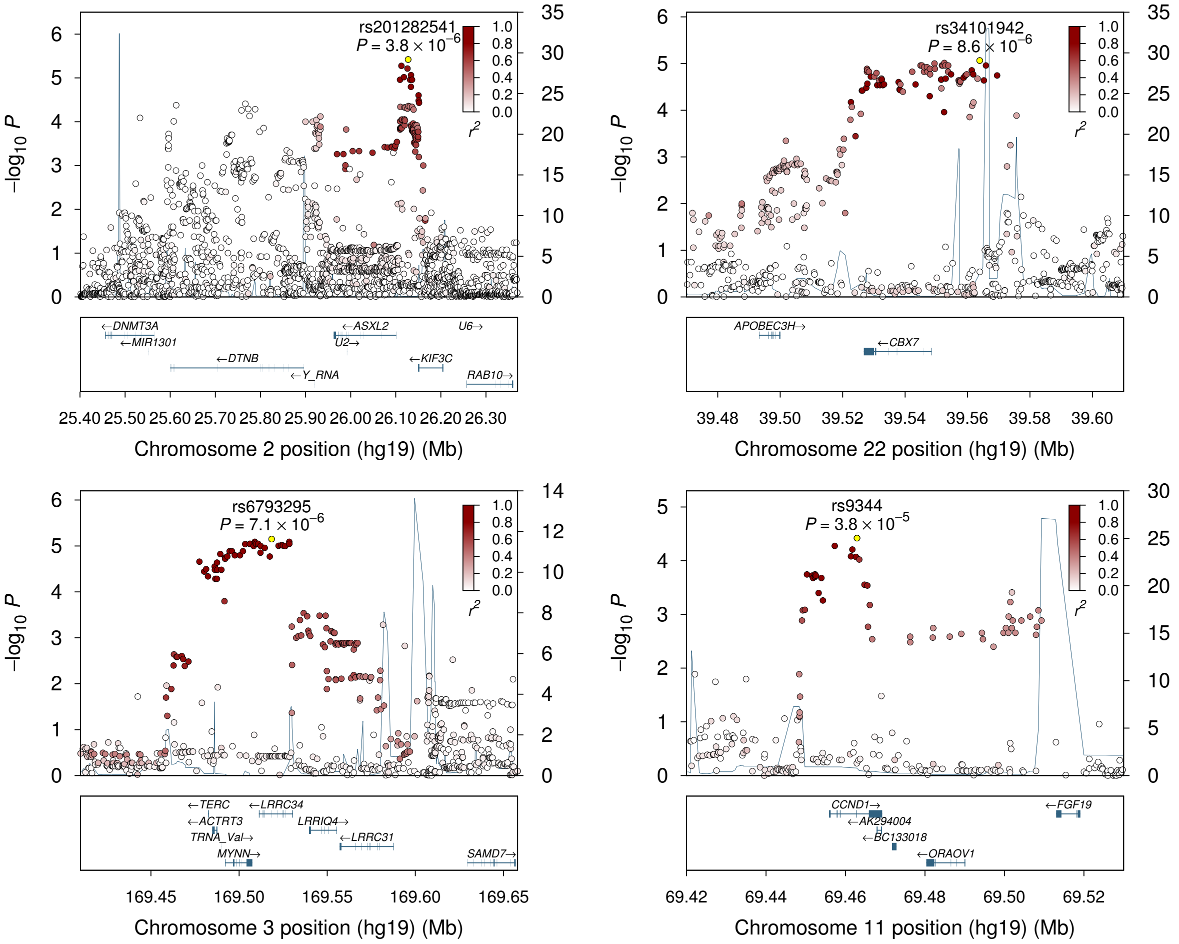

Association plots for MM risk loci identified in previous studies. **(a)** (pages 1 and 2): meta-analysis P values for the Swedish-Norwegian samples and the Icelandic MM samples. **(b)** (pages 3 and 4): meta-analysis P values for the Swedish-Norwegian samples and the Icelandic MM+MGUS samples.

Supplementary Figure 2

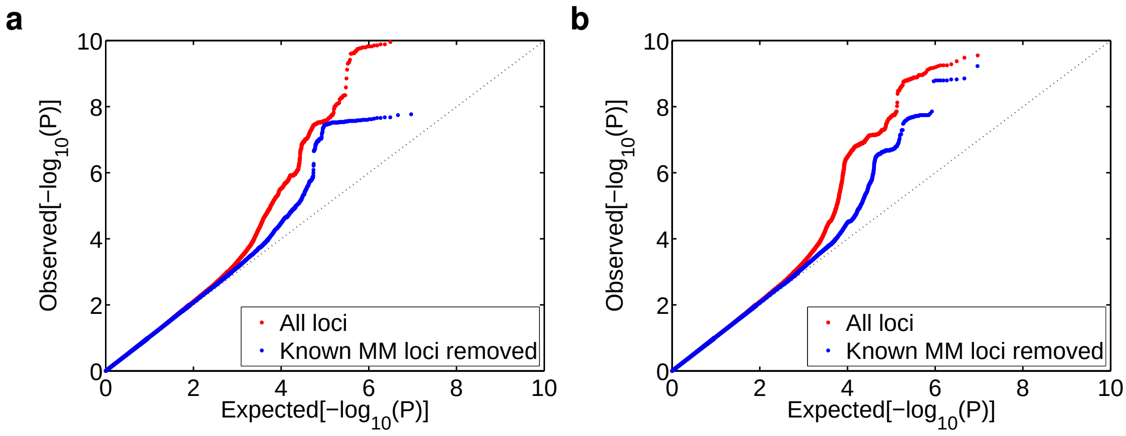

Quantile-quantile plots for the meta-analyses of the discovery sets: **(a)** Results obtained with the Swedish-Norwegian data and the Icelandic MM data; **(b)** Results obtained with the Swedish-Norwegian data and the Icelandic MM+MGUS data. A total of 12.1 million SNPs were used in the meta-analyses. Red: results for all SNPs. Blue: results after removing SNPs belonging to MM risk loci discovered in previous association studies (**Supplementary Fig. 1** and **Supplementary Table 1**). We observed minimal inflation of meta-analysis P values (genomic inflation factor λ=1.005 and 1.020 for the two meta-analyses, respectively).

Supplementary Figure 3

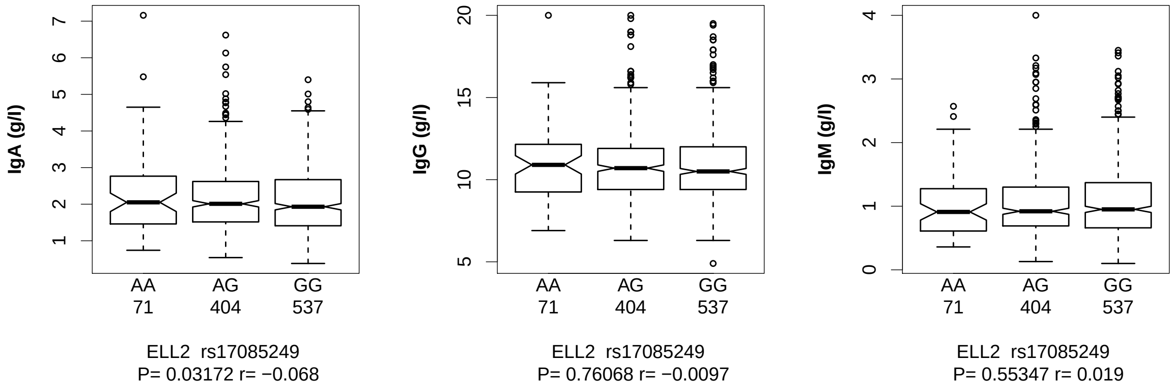

Blood concentrations of IgA, IgG and IgM values per genotype in 1,012 Swedish blood donors. These donors were genotyped previously for rs17085249, which is in tight linkage disequilibrium with the *ELL2* sentinel SNP rs56219066 (r^2=0.95). Boxes indicate medians and the first and third quartiles. Whiskers indicate first and third quartiles $\pm$ 1.5 times the interquartile range or the minimum/maximum values. Notches indicate confidence intervals around the median. Pearson correlation P values indicated.

Supplementary Table 1: Association results for known MM risk loci

Chr; Sentinel SNP	Candidate gene(s)	A1/A2[1]	Population	MM + MGUS			MM only			Prev. reported loci; LD values[2]
				RAF	Beta	*P*	RAF	Beta	*P*	
2p23.3 rs12986445	*DTNB, ASXL2*	A/T	Sweden/Norway	0.74	0.16	2.9×10^{-4}	0.74	0.16	2.9×10^{-4}	rs6746082; 0.08/0.005
			Iceland	0.75	0.199	5.1×10^{-3}	0.75	0.299	9.6×10^{-4}	
			**Combined**	**0.74**	**0.17**	**5.3×10^{-6}**	**0.74**	**0.18**	**2.6×10^{-6}**	
3p22.1 rs73071352	*ULK4*	A/G	Sweden/Norway	0.14	0.23	1.9×10^{-5}	0.14	0.23	1.9×10^{-5}	rs1052501; 1/0.81
			Iceland	0.12	0.30	3.4×10^{-4}	0.12	0.28	7.6×10^{-3}	
			**Combined**	**0.13**	**0.25**	**3.1×10^{-8}**	**0.14**	**0.24**	**5.2×10^{-7}**	
3q26.2 rs6793295	*MYNN, LRRC34*	T/C	Sweden/Norway	0.72	0.16	1.5×10^{-4}	0.72	0.16	1.5×10^{-4}	rs10936599; 1/0.92
			Iceland	0.78	0.18	1.6×10^{-2}	0.78	0.19	4.0×10^{-2}	
			**Combined**	**0.74**	**0.16**	**7.1×10^{-6}**	**0.73**	**0.165**	**1.7×10^{-5}**	
6p21.3 rs6919908	*PSORS1C2, HLA*	T/C	Sweden/Norway	0.21	0.26	1.2×10^{-8}	0.21	0.26	1.2×10^{-8}	rs2285803; 0.94/0.57
			Iceland	0.26	0.17	8.3×10^{-3}	0.26	0.22	8.2×10^{-3}	
			**Combined**	**0.23**	**0.23**	**6.3×10^{-10}**	**0.23**	**0.25**	**3.8×10^{-10}**	
7p15.3 rs57104699	*DNAH11, CDCA7L*	A/C	Sweden/Norway	0.66	0.19	1.4×10^{-6}	0.66	0.19	1.4×10^{-6}	rs4487645; 0.96/0.92
			Iceland	0.66	0.18	4.7×10^{-3}	0.66	0.22	7.3×10^{-3}	
			**Combined**	**0.66**	**0.19**	**2.3×10^{-8}**	**0.66**	**0.20**	**3.5×10^{-8}**	
11q13 rs9344	*CCND1*	A/G	Sweden/Norway	0.53	0.147	1.7×10^{-4}	0.53	0.147	1.7×10^{-4}	rs603965; N/A
			Iceland	0.53	0.105	7.8×10^{-2}	0.53	0.078	3.0×10^{-1}	
			**Combined**	**0.53**	**0.135**	**3.8×10^{-5}**	**0.53**	0.132	**1.3** $\times 10^{-4}$	
17p11.2 rs57968458	*TNFRSF13B*	A/G	Sweden/Norway	0.10	0.35	1.2×10^{-8}	0.10	0.35	1.2×10^{-8}	rs4273077; 0.85/0.39
			Iceland	0.08	0.28	5.6×10^{-3}	0.08	0.40	1.2×10^{-3}	
			**Combined**	**0.098**	**0.33**	**2.8×10^{-10}**	**0.098**	**0.36**	**5.7×10^{-11}**	
22q13.1 rs34101942	*CBX7*	G–/GA	Sweden/Norway	0.62	0.17	2.2×10^{-5}	0.62	0.17	2.2×10^{-5}	rs877529; N/A
			Iceland	0.62	0.10	1.0×10^{-1}	0.62	0.23	2.7×10^{-3}	
			**Combined**	**0.62**	**0.15**	**8.6×10^{-6}**	**0.62**	**0.18**	**2.8×10^{-7}**	

[1]Risk alleles underlined; [2]LD values are represented as D'/r^2 values between sentinel SNPs from this study and the previously reported loci. Abbreviations: RAF, risk allele frequency. Logistic regression and meta-analysis *P* values indicated.

Supplementary Table 2. Novel loci associating with MM or MM+MGUS at $P<5\times10^{-8}$

Populations	EAF	MM		MM+MGUS	
		OR (95%CI)	P value	OR (95%CI)	P value
**ELL2 - rs56219066-T**					
Sweden/Norway	0.732	1.20 (1.11-1.32)	3.8×10^{-5}	1.20 (1.11-1.32)	3.8×10^{-5}
Iceland	0.711	1.39 (1.17-1.64)	1.1×10^{-4}	1.32 (1.15-1.51)	3.9×10^{-5}
**Combined**		**1.23 (1.14-1.33)**	**6.5×10^{-8}**	**1.23 (1.15-1.33)**	**1.4×10^{-8}**
**TOM1 – rs138740-C**					
Sweden/Norway	0.364	1.20 (1.11–1.30)	2.4×10^{-6}	1.20 (1.11–1.30)	2.4×10^{-6}
Iceland	0.415	1.26 (1.09-1.46)	0.0017	1.15 (1.03-1.30)	0.015
**Combined**		**1.22 (1.13-1.30)**	**1.7×10^{-8}**	**1.19 (1.11-1.27)**	**1.3×10^{-7}**
**ARHGAP26 – rs74735889-T**					
Sweden/Norway	0.003	4.41 (2.53-7.67)	1.6×10^{-7}	4.41 (2.53-7.67)	1.6×10^{-7}
Iceland	0.002	2.33 (0.79-6.87)	0.12	3.51 (0.79-6.87)	8.4×10^{-4}
**Combined**		**3.85 (2.35-6.30)†**	**8.2×10^{-8}†**	**4.06 (2.61-6.33)†**	**5.9×10^{-10}†**

Association results for *ELL2* rs56219066, *TOM1* rs138740, and ARHGAP rs74735889 in the meta-analyses of the Swedish-Norwegian and Icelandic discovery samples. **†**By direct genotyping of the Swedish case and control samples the signal at 5q31 was no longer significant (OR=1.69; *P*=0.014). Abbreviations: EAF, effect allele frequency; OR, odds ratio. Logistic regression and meta-analysis P values indicated.
